# Effects of protein supplementation during pilates training on body composition, core muscle endurance, and joint flexibility in trained women: a randomized controlled trial

**DOI:** 10.1080/15502783.2025.2472891

**Published:** 2025-02-26

**Authors:** Christina Karpouzi, Antigoni Kypraiou, Vassilis Mougios, Anatoli Petridou

**Affiliations:** Aristotle University of Thessaloniki, Laboratory of Evaluation of Human Biological Performance, School of Physical Education and Sport Science at Thessaloniki, Thessaloniki, Greece

**Keywords:** Bioelectrical impedance analysis, Cadillac, dual-energy X-ray absorptiometry, physical performance, Reformer, whey protein

## Abstract

**Background:**

Pilates is a popular type of exercise, aimed at improving core muscle strength and endurance, core stability, and joint flexibility through a variety of whole-body exercises. Research has shown that Pilates improves body composition, muscle endurance, and joint flexibility. Adequate protein intake is a key factor in supporting the adaptive response of skeletal muscle to exercise training. However, whether protein supplementation augments the adaptations to Pilates training remains unknown. Thus, the aim of the present study was to investigate the effects of protein supplementation during Pilates training on body composition, core muscle endurance, and joint flexibility in trained women.

**Methods:**

Nineteen Pilates-trained women (31 ± 9 y) performed 10 weeks of Pilates training using the Reformer and Cadillac apparatuses, at least 2 times per week. Participants were randomly allocated to either a placebo (*n* = 10) or protein supplementation group (*n* = 9) in a quadruple-blind (participants, intervention providers, investigators, and outcome assessors) design. Participants received 0.6 g of maltodextrin or whey protein per kg body weight daily, respectively. Habitual dietary intake was monitored throughout the study. Before and after the intervention, anthropometric measures (body weight, body mass index, waist and hip circumferences), body composition [through full-scan dual-energy X-ray absorptiometry (DXA) and multifrequency bioelectrical impedance analysis (BIA)], core muscle endurance (through the McGill’s torso muscular endurance test battery), and joint flexibility (through the sit-and-reach test) were assessed. Data were analyzed by 2-way ANOVA (supplement × time) with repeated measures on time. Common DXA and BIA variables (whole-body fat and lean mass) were compared through paired Student’s t tests and subjected to Pearson’s correlation analysis. The level of statistical significance was set at α = 0.05.

**Results:**

Participants received, on average, 1.3 g protein/kg body weight/day from their habitual diet. After 10 weeks of Pilates training and regardless of supplementation, body fat (assessed by BIA) and hip circumference decreased; lean mass, total water, and extracellular water (by BIA) increased; and arm lean mass, trunk bone mineral content, and trunk bone area (by DXA) increased (all *p* < 0.05). The common BIA and DXA variables were highly correlated (*r* > 0.78, *p* < 0.001) and did not differ pre-intervention (*p* > 0.1), although they differed post-intervention (*p* < 0.001), with BIA overestimating lean mass compared with DXA. Core muscle endurance and joint flexibility increased with training (*p* < 0.05), with no effect of supplementation.

**Conclusion:**

Ten weeks of Pilates training improved core muscle endurance, joint flexibility, and aspects of body composition in healthy trained women, but these adaptations were not enhanced by daily supplementation with 0.6 g of protein per kilogram body weight.

## GRAPHICAL ABSTRACT

**Figure:**
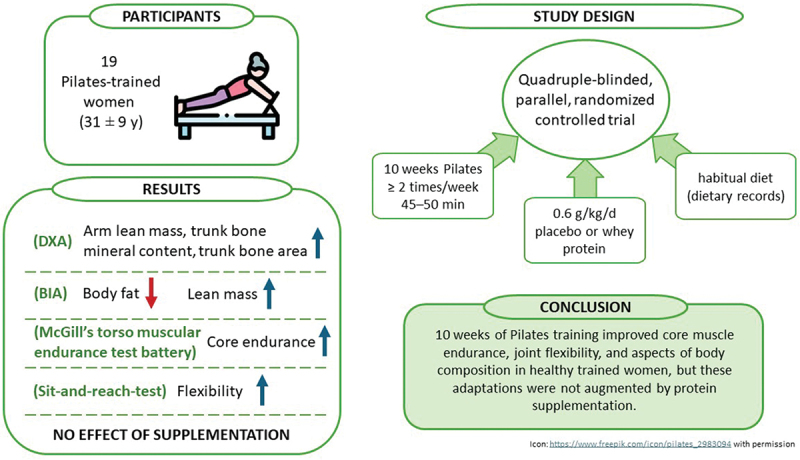

## 1. Introduction

Pilates is a popular type of exercise, developed by Joseph Pilates in 1920 and aimed at improving core muscle strength and endurance, core stability, and joint flexibility through a variety of whole-body exercises requiring controlled movements and consistent breathing [1,2]. Pilates training can be performed on the ground, using portable equipment, or on specialized apparatuses such as the Reformer and Cadillac [2]. Training load can be adjusted to individual needs and characteristics, making the Pilates method a feasible and tolerated type of exercise for almost all people, regardless of age and health status [3,4].

Research has shown that Pilates training improves body composition [5–20] and physical performance [6,12,15,18,21–32] in various population groups. Specifically, Pilates training lasting 6 or more weeks has been reported to decrease body fat [5–7,9–17] and increase lean body mass [5,9–11,17–19], core muscle strength and endurance [6,12,15,18,21,23–27], as well as joint flexibility [6,12,15,22–25,28–32] in young, middle-aged, and older adults.

It is well established that nutrition plays a crucial role in supporting adaptations to exercise training [33]. In particular, adequate protein intake is essential for muscle tissue reconditioning following exercise [34]. This is mainly because protein-derived amino acids serve as substrates for muscle protein synthesis and regulate cellular processes that are responsible for adaptive responses to training [34,35]. For these reasons, protein supplements are widely used among recreational exercisers as well as competitive athletes [36,37]. There is now ample evidence that protein supplementation during resistance training (lasting at least 6 weeks) improves body composition and increases lean body mass, muscle strength, and physical/functional performance in healthy adults [38–40]. Additionally, protein supplementation has been reported to further increase endurance capacity, lean body mass, and physical performance when combined with endurance training in healthy and clinical populations [41].

It should be noted that most of the studies on the effectiveness of protein supplementation during training have focused on resistance or endurance exercise modalities. The fact that Pilates training results in several physiological adaptations [8,20,22] for which protein-derived amino acids are a prerequisite [35] begets the hypothesis that protein supplementation augments the adaptations to Pilates training. To our knowledge, this hypothesis has not been tested. Nevertheless, numerous popular websites recommend protein supplements to Pilates exercisers. Therefore, the aim of the present study was to investigate the effects of protein supplementation (compared to placebo) during 10 weeks of Pilates training on body composition, core muscle endurance, and joint flexibility in healthy trained women.

## 2. Materials and methods

### 2.1. Participants

The sample size was calculated a priori using the G*Power software (version 3.1.9.2, Kiel University, Kiel, Germany). To detect significant effects with a medium effect size of 0.059 (as η^2^ for factorial ANOVA, [42]), α of 0.05, power of 0.8, and correlation coefficient between repeated measures of 0.8 (based on similar studies in our laboratory), a sample size of 16 was required. To account for possible dropouts, 20 participants were recruited by word of mouth from among customers of fitness clubs in the area of Thessaloniki, Greece, between November 2022 and January 2023.

The inclusion criteria were: (i) female sex, (ii) age 18 to 55, (iii) regular menstrual cycle, (iv) regular Pilates training (at least 2 times a week, 50 min each session, for the past 4 months), as assessed by gym records or questionnaires, and (v) mixed isoenergetic diet for the past 4 months. The exclusion criteria were: (i) musculoskeletal injuries that could interfere with training, (ii) chronic disease, (iii) milk allergy (because the protein supplement was whey protein), (iv) pregnancy, lactation, or planning a pregnancy during the study, and (v) regular use of prescription medicine or of supplements that could affect muscle function or recovery over the past month.

### 2.2. Ethics

The participants received detailed written and oral information about the purpose, procedures, and possible risks of the study, after which they gave written informed consent to participate. The study was approved by the Ethics Committee of the School of Physical Education and Sport Science at Thessaloniki, Aristotle University of Thessaloniki (approval number 139/11 January 2023), was carried out according to the Declaration of Helsinki, and was registered at clinicaltrials.gov, NCT05849350.

### 2.3. Experimental design

A quadruple-blind (participants, intervention providers, investigators, and outcome assessors), randomized controlled trial with a parallel-group design was employed to determine the effect of protein supplementation during 10 weeks of Pilates training on body composition, core muscle endurance, and joint flexibility. Following inclusion, participants were randomly (by drawing lots) allocated to either a placebo or protein group in a 1:1 ratio. During the week preceding the onset of the intervention and the week following the end of the intervention, anthropometric measures, body composition, core muscle endurance, and joint flexibility were assessed by the same assessor. The study was conducted between January and April 2023.

### 2.4. Pilates training protocol

Pilates sessions were supervised by certified Pilates instructors and were carried out at four well-equipped commercial gyms using the Reformer and Cadillac apparatuses (CoreMotion, Athens, Greece, and Alpha Pilates, Lamia, Greece, respectively). Each session lasted 50 minutes and was divided into three phases: warm-up (5 to 7 minutes), main phase (40 minutes), and cool-down (3 to 5 minutes). Warm-up consisted of hip mobility, pelvic stability, and core engagement exercises, while cool-down consisted of static stretching. The exercises performed during the main phase were based on the classical series of the Pilates method (foot work, hip work, spinal articulation, abdominal, up-stretch, knee stretch, pulling straps series, etc.) [43,44]. Since the participants were Pilates trained, the level of the exercises was intermediate, including more challenging variations of the common exercises from both the classical and contemporary repertoires, such as in training sessions found online [45], depending on the level of each participant.

The intensity of Pilates training was estimated on the basis of heart rate measurements using telematic sensors and software (Polar Team Pro GPS telematic system, Polar, Kempele, Finland) during a randomly selected training session. The participants trained for 10 weeks, at least 2 times per week, in groups of up to 5, supervised by an instructor with an average experience of 5 years and certified by the StudioOne Sport and Fitness School, which is accredited by the European Register of Exercise Professionals. The number of Pilates training sessions attended by each participant was recorded in the gyms᾽ customer database. Participants were asked to refrain from any other type of exercise during the study.

### 2.5. Supplementation

During the 10-week training period, the participants of the placebo group consumed 0.6 g of maltodextrin per kilogram of body weight per day (equivalent to the carbohydrate content of a large banana), while the participants of the protein group consumed 0.6 g of whey protein per kilogram of body weight per day. Both supplements were supplied in powder form by Warriorlab, Athens, Greece. The supplements were taken in two daily doses of 0.3 g/kg body weight, each providing 1.2 kcal/kg body weight, and placed 12 hours apart. The adherence to supplementation was assessed by having the subjects complete a supplement intake logbook.

### 2.6. Anthropometric measurements

Body weight was measured using a digital scale (Smart Body Fat Scale, Huawei, Tokyo, Japan) to the nearest 0.1 kg with minimal clothing. Body height was measured with a stadiometer to the nearest 0.01 m for the calculation of body mass index (BMI). Waist and hip circumferences were measured using a non-extendable tape to the nearest 0.5 cm according to the World Health Organization STEPwise Approach to Surveillance protocol [46].

### 2.7. Assessment of body composition

Body composition was assessed through two methods: (i) full-scan dual-energy X-ray absorptiometry (DXA) in a GE Lunar iDXA-ME scanner (General Electric, Boston, MA) to measure whole-body and regional (arms, legs, and trunk) bone mineral density (BMD), bone mineral content (BMC), bone area, fat (including visceral, subcutaneous, and intramuscular fat), lean (total minus fat), and lean soft (total minus fat minus BMC) and (ii) multifrequency bioelectrical impedance analysis (BIA) with a Bodystat Quadscan 4000 apparatus (Douglas, Isle of Man, British Isles) to measure fat, lean, water, extracellular water (ECW), and intracellular water (ICW), all referring to the whole body. The participants were asked to abstain from food and liquid consumption for 3 hours and refrain from any kind of exercise for 12 hours before the measurements. During the measurements, they lay still on an examination bed, with arms away from the trunk and legs apart.

### 2.8. Assessment of core muscle endurance

Core muscle endurance was assessed through the McGill’s torso muscular endurance test battery [47], which consists of trunk flexor, trunk lateral (right and left), and trunk extensor endurance tests. In each test, the participant had to hold a static position without support for as long as possible. In the trunk flexor test, the participant held the sit-up position with the trunk at a 60° incline, and knees and hips bent at 90°. In the trunk lateral tests, the participant held a full side-bridge position, keeping legs extended, hips elevated, and the body in straight alignment, with support from the feet and the forearm of the lower arm, while keeping the upper arm on the side. In the trunk extensor test, the participant held a horizontal position while lying prone on a treatment table, with the upper part of the body (above the iliac crests) extending from the table and the lower part secured by the investigator. Time in each test was recorded with an electronic stopwatch to the nearest second until the participant could no longer hold the proper position. Participants were asked to refrain from strenuous exercise for 24 hours before the assessment.

### 2.9. Assessment of joint flexibility

Joint flexibility was assessed by the sit-and-reach test, which was performed according to the guidelines of the American College of Sports Medicine [48]. The participant sat, with her bare feet resting against a sit-and-reach box. The investigator stabilized her knees and asked her to keep them extended. The participant then positioned one hand on top of the other and leaned forward as far as possible along the measurement scale. The farthest distance reached was recorded. The average value of two trials, spaced 10 s apart, was documented. Participants were asked to refrain from strenuous exercise for 24 hours before the test.

### 2.10. Dietary control

The participants were asked not to modify their diet during the study and to record their food intake for 3 days (2 weekdays and 1 weekend day) on the first, fifth, and final week of the intervention. Dietary records were analyzed in Microsoft Excel using the FoodData Central database provided by the US Department of Agriculture [49].

### 2.11. Monitoring of menstrual status

The participants were asked to note the date of onset of menses just before and during the intervention period. This information was used to calculate the day of the cycle when each pre- and post-intervention assessment was performed.

### 2.12. Motivation strategy

One of the investigators contacted the participants weekly throughout the intervention period to motivate them to adhere to their training and supplementation schedules, encourage them to maintain their habitual dietary intake, and inquire about any adverse events.

### 2.13. Statistical analysis

Data are expressed as the mean ± standard deviation (SD). Two-way mixed analysis of variance (ANOVA), with supplement (placebo or protein) as the between-subject factor and time (pre- and post-intervention) as the within-subject factor, was performed to compare anthropometric characteristics, body composition, core muscle endurance, joint flexibility, and menstrual variables between groups over time. Differences in energy and macronutrient intakes were also examined by two-way mixed ANOVA, except that there were three levels of time (first, fifth, and last week of the intervention). Effect sizes (*ES*) for main effects and interactions were determined as partial η^2^ and were classified as small (0.01–0.058), medium (0.059–0.137), or large (>0.137), according to Cohen [42]. Age and attendance of training sessions were compared between groups through independent Student’s *t* test. Common DXA and BIA variables (that is, whole-body fat and lean mass) were compared through paired Student’s *t* tests and subjected to Pearson’s correlation analysis. The level of statistical significance was set at α = 0.05. All statistical analyses were conducted using the SPSS version 29.0 (SPSS, Chicago, IL, USA).

## 3. Results

One participant left the study for health-related reasons before the baseline assessment. Thus, the study was conducted with 19 participants, aged 31 ± 9 y. At the end, unblinding revealed that 10 participants (aged 33 ± 11 y) had taken placebo, while 9 (aged 29 ± 4 y) had taken protein, with no significant difference in age between groups (*p* = 0.328). The adherence to supplementation was 90 ± 11% and 86 ± 19% in the placebo and protein groups, respectively. The number of Pilates sessions attended during the 10-week intervention was 26 ± 5 and 23 ± 5, respectively, with no significant difference between groups (*p* = 0.287). The average intensity of Pilates training was 55% of the maximal heart rate (calculated as 220 – age).

### 3.1. Anthropometric characteristics

Table 1 presents the anthropometric characteristics of the participants pre- and post-intervention. Following 10 weeks of Pilates training, hip circumference decreased significantly by 2.5 ± 4.1 cm, regardless of supplementation. Body weight, BMI, and waist circumference exhibited a main effect of time (increases in body weight and BMI, as opposed to a decrease in waist circumference) that approached statistical significance (*p* between 0.056 and 0.066), with large *ES* (0.185 to 0.198).

**Table 1.** Anthropometric characteristics (mean ± SD) of the participants before and after 10 weeks of Pilates training with placebo or protein supplementation.

Parameter	Placebo group (*n* = 10)		Protein group (*n* = 9)		Supplement × time interaction		Main effect of supplement		Main effect of time	
	Pre	Post	Pre	Post	*p*	*ES*	*p*	*ES*	*p*	*ES*
Body weight (kg)	61.4 ± 7.7	62.4 ± 8.6	60.5 ± 9.5	60.9 ± 8.6	0.465	0.032	0.761	0.006	0.066	0.185
Body mass index (kg/m^2^)	21.6 ± 2.8	22.0 ± 3.2	22.1 ± 3.4	22.2 ± 3.1	0.457	0.033	0.808	0.004	0.056	0.198
Waist circumference (cm)	75.3 ± 8.9	74.1 ± 7.1	73.8 ± 9.7	70.3 ± 4.1	0.350	0.052	0.453	0.033	0.063	0.189
Hip circumference (cm)	95.9 ± 6.9	95.1 ± 7.0	96.9 ± 7.4	92.6 ± 10.0	0.059	0.194	0.839	0.002	**0.010**	0.329

*ES*, effect size (as partial η^2^) following 2-way ANOVA. Boldface indicates significant outcomes (*p* < 0.05).

### 3.2. Body composition

Table 2 shows the body composition variables of the two groups pre- and post-intervention, as assessed with DXA. Most of the variables did not exhibit any statistically significant outcomes. We only found four significant effects of time, that is, increases in arm lean mass, arm lean soft mass, trunk BMC, and trunk bone area (*p* < 0.05), all with large *ES* (0.213 to 0.293). Also, the interaction of supplement and time in leg fat mass approached statistical significance (*p* = 0.054) with large *ES* (0.202), due to an increase in the placebo group, as opposed to no change in the protein group.

**Table 2.** Body composition parameters (mean ± SD), as assessed with full-scan dual-energy X-ray absorptiometry, before and after 10 weeks of Pilates training with placebo or protein supplementation.

Parameter	Placebo group (*n* = 10)		Protein group (*n* = 9)		Supplement × time interaction		Main effect of supplement		Main effect of time	
	Pre	Post	Pre	Post	*p*	*ES*	*p*	*ES*	*p*	*ES*
*Whole body*										
BMD (g/cm^2^)	1.119 ± 0.077	1.122 ± 0.065	1.145 ± 0.065	1.144 ± 0.054	0.823	0.003	0.417	0.039	0.870	0.002
BMC (g)	2382 ± 255	2383 ± 265	2395 ± 150	2408 ± 134	0.229	0.084	0.846	0.002	0.166	0.109
Bone area (cm^2^)	2127 ± 151	2120 ± 168	2093 ± 93	2105 ± 79	0.591	0.017	0.672	0.011	0.874	0.002
Fat (kg)	18.8 ± 6.9	19.3 ± 7.3	19.4 ± 6.1	19.5 ± 5.8	0.253	0.076	0.907	0.001	0.151	0.117
Fat (%)	30.3 ± 6.5	30.8 ± 7.1	31.8 ± 5.8	31.7 ± 5.2	0.314	0.059	0.685	0.010	0.489	0.029
Lean (kg)	42.2 ± 3.7	42.3 ± 3.9	40.7 ± 3.8	41.1 ± 3.6	0.330	0.056	0.434	0.036	0.105	0.147
Lean (%)	69.7 ± 6.5	69.2 ± 7.1	68.2 ± 5.8	68.3 ± 5.2	0.331	0.056	0.682	0.010	0.495	0.028
Lean soft (kg)	39.8 ± 3.5	39.9 ± 3.7	38.3 ± 3.7	38.7 ± 3.5	0.356	0.050	0.408	0.041	0.119	0.137
Lean soft (%)	65.8 ± 6.2	65.3 ± 6.7	64.2 ± 5.6	64.3 ± 4.9	0.347	0.052	0.636	0.013	0.549	0.022
*Arms*										
BMD (g/cm^2^)	0.744 ± 0.108	0.771 ± 0.117	0.756 ± 0.127	0.770 ± 0.132	0.853	0.002	0.898	0.001	0.566	0.020
BMC (g)	307 ± 39	307 ± 41	306 ± 36	310 ± 39	0.484	0.029	0.944	0.000	0.363	0.049
Bone area (cm^2^)	419 ± 68	407 ± 77	411 ± 52	409 ± 63	0.763	0.005	0.913	0.001	0.711	0.008
Fat (kg)	2.2 ± 1.0	2.3 ± 0.9	2.3 ± 0.8	2.3 ± 0.7	0.965	0.000	0.839	0.003	0.229	0.084
Fat (%)	32.4 ± 7.6	32.8 ± 7.7	34.5 ± 7.0	34.6 ± 6.1	0.711	0.008	0.559	0.020	0.582	0.018
Lean (kg)	4.4 ± 0.5	4.5 ± 0.6	4.3 ± 0.7	4.3 ± 0.6	0.758	0.006	0.580	0.018	**0.041**	0.224
Lean (%)	67.6 ± 7.6	67.2 ± 7.7	65.5 ± 7.0	65.4 ± 6.1	0.711	0.008	0.559	0.020	0.582	0.018
Lean soft (kg)	4.1 ± 0.5	4.2 ± 0.6	4.0 ± 0.7	4.0 ± 0.6	0.799	0.004	0.559	0.020	**0.047**	0.213
Lean soft (%)	62.9 ± 7.1	62.6 ± 7.2	60.8 ± 6.7	60.7 ± 5.9	0.746	0.006	0.523	0.024	0.671	0.011
*Legs*										
BMD (g/cm^2^)	1.149 ± 0.089	1.140 ± 0.072	1.158 ± 0.081	1.160 ± 0.085	0.215	0.089	0.703	0.009	0.392	0.043
BMC (g)	841 ± 96	841 ± 99	837 ± 72	836 ± 66	0.929	0.000	0.921	0.001	0.815	0.003
Bone area (cm^2^)	731 ± 48	736 ± 52	726 ± 37	721 ± 36	0.165	0.110	0.645	0.013	0.944	0.000
Fat (kg)	7.5 ± 2.6	7.8 ± 2.6	7.8 ± 1.8	7.8 ± 1.8	0.054	0.202	0.871	0.002	0.234	0.082
Fat (%)	34.3 ± 5.6	34.9 ± 5.7	35.9 ± 4.5	35.8 ± 3.6	0.246	0.078	0.581	0.018	0.421	0.038
Lean (kg)	14.1 ± 1.3	14.1 ± 1.4	13.8 ± 1.7	13.8 ± 1.8	0.963	0.000	0.703	0.009	0.914	0.001
Lean (%)	65.7 ± 5.6	65.1 ± 5.7	64.1 ± 4.5	64.2 ± 3.6	0.246	0.078	0.581	0.018	0.421	0.038
Lean soft (kg)	13.2 ± 1.2	13.2 ± 1.3	13.0 ± 1.7	13.0 ± 1.7	0.965	0.000	0.697	0.009	0.908	0.001
Lean soft (%)	61.8 ± 5.3	61.2 ± 5.4	60.2 ± 4.3	60.3 ± 3.4	0.285	0.067	0.558	0.021	0.456	0.033
*Trunk*										
BMD (g/cm^2^)	0.951 ± 0.078	0.952 ± 0.073	0.995 ± 0.057	0.986 ± 0.048	0.151	0.117	0.209	0.091	0.327	0.057
BMC (g)	712 ± 84	718 ± 87	731 ± 72	744 ± 71	0.390	0.044	0.530	0.024	**0.030**	0.248
Bone area (cm^2^)	747 ± 50	752 ± 53	734 ± 49	754 ± 43	0.114	0.140	0.799	0.004	**0.017**	0.293
Fat (kg)	8.3 ± 3.5	8.5 ± 4.0	8.5 ± 3.7	8.6 ± 3.5	0.483	0.029	0.940	0.000	0.241	0.080
Fat (%)	28.4 ± 8.2	28.8 ± 9.1	29.7 ± 8.2	29.6 ± 7.7	0.553	0.021	0.788	0.004	0.648	0.013
Lean (kg)	20.1 ± 2.2	20.2 ± 2.1	19.2 ± 1.5	19.5 ± 1.4	0.329	0.056	0.346	0.052	0.092	0.158
Lean (%)	71. 6 ± 8.2	71.2 ± 9.1	70.3 ± 8.2	70.4 ± 7.7	0.553	0.021	0.788	0.004	0.648	0.013
Lean soft (kg)	19.4 ± 2.1	19.5 ± 2.1	18.5 ± 1.5	18.8 ± 1.4	0.351	0.051	0.322	0.058	0.111	0.142
Lean soft (%)	69.0 ± 8.0	68.7 ± 8.8	67.6 ± 8.1	67.7 ± 7.6	0.561	0.020	0.753	0.006	0.654	0.012

BMC, bone mineral content; BMD, bone mineral density; *ES*, effect size (as partial η^2^) following 2-way ANOVA. Boldface indicates significant outcomes (*p* < 0.05).

Body composition variables measured by BIA exhibited only main effects of time (Table 3). Specifically, body fat decreased (*p* < 0.05), whereas lean mass, total water, and ECW increased (*p* ≤0.001), all with large *ES* (0.297 to 0.757). To explore the discrepancy between these findings and the outcomes of the DXA analysis (in which there were no significant effects of time on whole-body fat or lean tissue), we compared the fat and lean tissue values (in terms of both mass and percentage) of all the participants (*n* = 19) between the two methods. We found that, although there was no significant difference in any of these variables at baseline (*p* > 0.1), all variables differed significantly post-intervention (*p* < 0.001), with BIA overestimating lean mass by 3.0 ± 1.6 kg, compared with DXA. The BIA variables were highly correlated with the corresponding DXA variables, with correlation coefficients ranging from 0.781 to 0.977 and with *p* < 0.001.

**Table 3.** Body composition parameters (mean ± SD), as assessed with bioelectrical impedance analysis in the whole body, before and after 10 weeks of pilates training with placebo or protein supplementation.

Parameter	Placebo group (*n* = 10)		Protein group (*n* = 9)		Supplement × time interaction		Main effect of supplement		Main effect of time	
	Pre	Post	Pre	Post	*p*	*ES*	*p*	*ES*	*p*	*ES*
Fat (kg)	18.4 ± 5.1	17.1 ± 7.4	19.1 ± 6.8	17.0 ± 5.6	0.518	0.025	0.917	0.001	**0.016**	0.297
Fat (%)	29.5 ± 4.6	26.7 ± 7.4	30.9 ± 5.4	27.4 ± 4.8	0.712	0.008	0.681	0.010	**0.001**	0.466
Lean (kg)	43.1 ± 3.3	45.3 ± 3.6	41.4 ± 2.9	44.0 ± 3.8	0.749	0.006	0.326	0.057	**<0.001**	0.624
Lean (%)	70.5 ± 4.6	73.3 ± 7.4	69.1 ± 5.4	72.6 ± 4.8	0.712	0.008	0.681	0.010	**0.001**	0.466
Water (L)	30.0 ± 2.3	32.1 ± 2.2	28.7 ± 1.8	30.9 ± 2.3	0.914	0.001	0.184	0.101	**<0.001**	0.620
Water (%)	49.1 ± 3.1	51.9 ± 4.7	48.0 ± 5.2	51.1 ± 4.0	0.886	0.001	0.611	0.016	**<0.001**	0.486
Extracellular water (L)	13.7 ± 1.3	15.0 ± 1.1	13.1 ± 1.1	14.4 ± 1.1	0.998	0.000	0.268	0.072	**<0.001**	0.757
Extracellular water (%)	22.4 ± 1.7	24.2 ± 2.0	21.9 ± 2.8	23.8 ± 1.6	0.925	0.001	0.650	0.012	**<0.001**	0.672
Intracellular water (L)	16.6 ± 2.3	16.8 ± 1.5	15.9 ± 1.8	16.2 ± 1.7	0.927	0.001	0.412	0.040	0.650	0.012
Intracellular water (%)	27.2 ± 3.0	27.0 ± 1.7	26.5 ± 2.0	26.6 ± 1.5	0.858	0.002	0.501	0.027	0.972	0.000

*ES*, effect size (as partial η^2^) following 2-way ANOVA. Boldface indicates significant outcomes (*p* < 0.05).

### 3.3. Core muscle endurance

**Figure 1.**
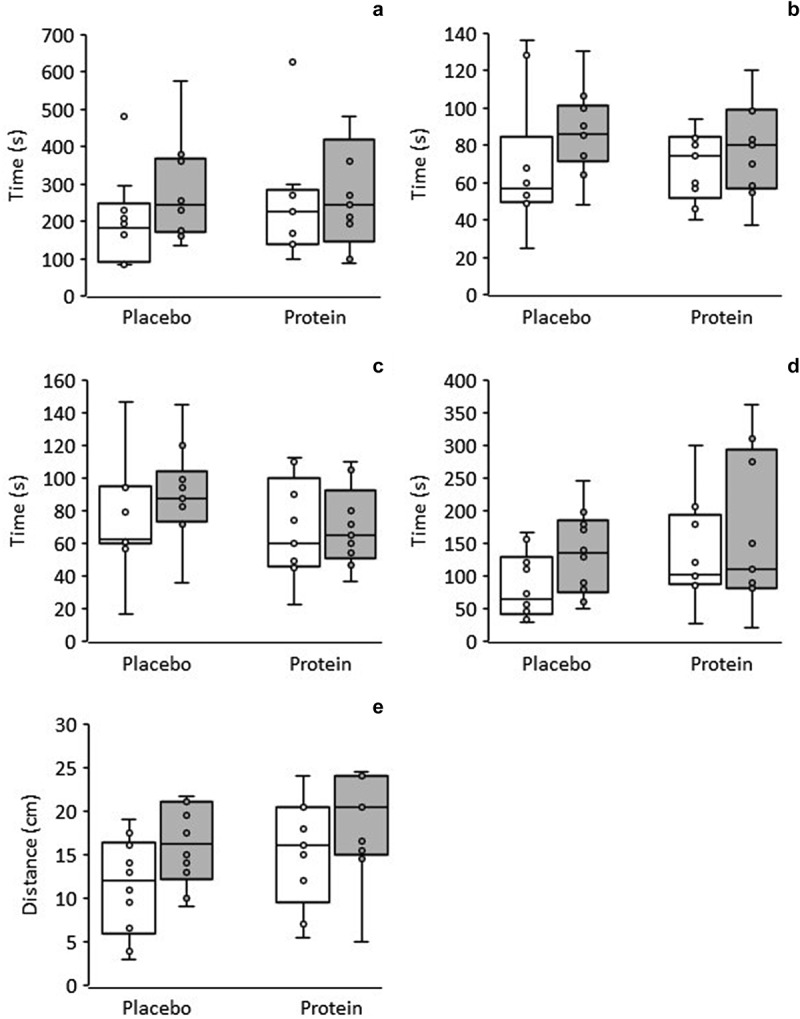
Box plots of performance in the McGill’s torso muscular endurance tests of trunk flexor (a), trunk lateral right (b), trunk lateral left (c), and trunk extensor (d), as well as of performance in the sit-and reach test (e), pre- (white boxes) and post-intervention (gray boxes) in the placebo and protein groups. Each box represents the interquartile range, and the center line represents the median. Whiskers are extended to the most extreme data point that is no more than 1.5 times the interquartile range from the edge of the box (Tukey style). Dots represent individual values.

Two-way ANOVA produced significant main effects of time on all four components of the McGill’s torso muscular endurance test battery, that is, the trunk flexor (*p* = 0.035, *ES* = 0.235), trunk lateral right (*p* = 0.041, *ES* = 0.224), trunk lateral left (*p* = 0.036, *ES* = 0.233), and trunk extensor endurance tests (*p* < 0.001, *ES* = 0.519), but no significant interaction or main effect of supplement. Performance improved in all tests by an average 23 to 48%; this is depicted in Figure 1a-d in the form of box plots to allow a clearer visualization of interindividual variability.

### 3.4. Joint flexibility

Performance in the sit-and-reach test (Figure 1e) exhibited a significant main effect of time (*p* < 0.001; *ES* = 0.489) but no significant interaction or main effect of supplement. The distance reached by the participants increased from 13.3 ± 6.1 cm at baseline to 17.2 ± 5.5 cm at the end of the intervention.

### 3.5. Habitual dietary intake

No significant difference was found between groups in the habitual energy or macronutrient intake, as assessed by the dietary analysis of the 3-day food records completed by the participants on the first, fifth, and tenth weeks of the intervention. Values from this 9-day dietary analysis were, for the placebo and protein groups, respectively: Daily energy intake, 1759 ± 292 and 1563 ± 203 kcal; carbohydrate, 40.1 ± 5.0 and 41.3 ± 4.6 % energy; fat, 39.1 ± 2.9 and 38.7 ± 3.6 % energy; and protein, 18.7 ± 3.5 and 19.5 ± 3.5 % energy, or 1.34 ± 0.31 and 1.26 ± 0.24 g/kg body weight. Alcohol intake differed between the placebo and protein groups, accounting for 2.0 ± 1.4 and 0.4 ± 0.7 % of energy, respectively (*p* = 0.006). The primary source of dietary protein was dairy products, especially full-fat cheese like gouda and feta. Other common sources were, in descending order, lean meat (turkey ham and chicken), red meat (beef and pork), nuts, eggs, and fish (tuna and seafood). With supplementation (adjusted for adherence), the daily protein intake of the protein group increased to 1.78 ± 0.28 g/kg body weight.

### 3.6. Menstrual status

There was no significant difference between groups, difference between the pre- and post-intervention evaluations, or interaction of supplement and time regarding the day of the cycle on which the participants were evaluated (*p* > 0.3).

### 3.7. Adverse events

During the intervention, the following adverse events were reported: bloating (by one participant in the placebo group and three in the protein group), nausea (by one participant in each group), constipation (by one participant in the protein group), facial rash (by one participant in the placebo group), and pyelonephritis (by one participant in the protein group).

### 3.8. Correlation between repeated measures

The average correlation coefficient between repeated measures for all the body composition and performance variables was 0.87, that is, higher than the assumed value of 0.8 used in the a priori calculation of sample size. Based on the value of 0.87 and the actual sample size (19 participants), the study was sufficiently powered to detect significant effects with an effect size (η^2^) as low as 0.032 (small)

## 4. Discussion

The present study investigated the effects of protein supplementation during 10 weeks of Pilates training on body composition, core muscle endurance, and joint flexibility in healthy trained women. Our main findings are that 10 weeks of Pilates training using the Reformer and Cadillac apparatuses (i) increased arm lean mass, trunk BMC, and trunk bone area, as assessed by DXA, (ii) decreased body fat and increased lean mass, total water, and ECW, as assessed by BIA, and (iii) increased core muscle endurance and joint flexibility. However, protein supplementation, which increased the average daily protein intake from 1.26 to 1.78 g/kg body weight, did not affect any outcome measure.

The absence of a significant change in the participants’ body weight after the intervention shows that they followed the suggestion not to modify their diet during the study. Our findings of decreased waist and hip circumferences after 10 weeks of Pilates training, both with large effect sizes, are in agreement with previous reports [5–7], although Gracia-Pastor et al. [14] found no change, Rogers and Gibson [15] found mixed results (decrease in waist circumference and no change in hip circumference), and Nasiri et al. [21] found no change in waist circumference (without examining hip circumference). These discrepancies in the literature may be related to differences in the concomitant changes in body weight and/or fat.

When assessing the effects of Pilates training on body composition, we found different results depending on the method used, that is, a decrease in body fat and increase in lean body mass with BIA, as opposed to only an increase in arm lean mass with DXA. These findings agree with most of the adequately controlled studies that examined the effects of Pilates training on body composition. Indeed, of the studies that used BIA, seven reported decreases in body fat (as either mass or percentage body mass) with Pilates training [5,7,9–13], whereas three found no change [18,23,50]. Similarly, of the studies that used skinfold thickness measurement, four reported decreases in body fat [14–17], whereas two found no change [21,24]. In contrast, of the few studies that used DXA, only one found a decrease in body fat with Pilates training [6], whereas three found no change [19,25,51]. Findings regarding lean body mass are mixed, with no clear differences between methods and with seven studies showing increases [5,9–11,17–19], seven showing no change [7,12,13,23,25,50,51], and one reporting a decrease [6].

The reason for the discrepancy between the measurements from DXA and those from BIA and skinfold thickness measurement regarding the effect of Pilates training on body composition is not clear. Notably, our study is the only one that used both DXA and BIA, showing good agreement between methods in the pre-intervention measures but remarkable differences in the post-intervention measures. This is probably due to the post-intervention increase in body water, which resulted in increased estimate of lean body mass by BIA. Thus, the absence of change in body composition, as assessed with the reference method of DXA [52], conflicts with the increase in body water, as assessed with BIA. We have no explanation for this discrepancy.

Regarding bone variables, the observed increases in trunk BMC and trunk bone area with Pilates training are potentially positive health outcomes. These increases may be due to the mechanical forces generated by muscle activity of the deep core and respiratory muscles, such as the transversus abdominis, internal oblique muscles, internal and external intercostal muscles, and diaphragm [2]. The absence of a significant change in trunk BMD is probably due to the increases in both BMC and bone area, since BMD is calculated as the ratio of BMC to bone area.

Our findings of increased core muscle endurance with Pilates training agree with those of most of the literature regarding trunk flexor endurance [6,12,15,18,21,23–26], trunk lateral endurance [23], and trunk extensor endurance [6,15,23,27]. Two studies have found no change in trunk flexor endurance [27,53], and one no change in trunk extensor endurance [53]. Likewise, our finding of increased joint flexibility agrees with the findings of the overwhelming majority of the relevant studies [6,12,15,23–25,28–32], with only two studies showing no change [21,50].

Several meta-analyses have shown that protein supplementation augments adaptations to resistance [38–40] and endurance training [41]. Pilates is a type of exercise that combines resistance and endurance elements. Hence, one would expect protein supplementation to benefit adaptations to Pilates training. Nevertheless, in the present study we show, for the first time to our knowledge, that, although Pilates training improved body composition, core muscle endurance, and joint flexibility of trained women, protein supplementation did not elicit any further improvements. One would expect that an increase in daily protein intake from 1.26 to 1.78 g/kg body weight would be sufficient to enhance the benefits of Pilates training on the outcome measures of the study, based on the recommendation for a daily protein intake of at least 1.6 g/kg body weight in resistance training [38,40] (as there is no such recommendation for Pilates training). Thus, our findings suggest that a daily protein intake of approximately 1.3 g/kg body weight is sufficient to support adaptations to Pilates training.

A limitation of the present study is that habitual dietary intake was assessed through 3-day dietary records kept by the participants themselves, which introduces the usual risk of misreporting. To mitigate this risk, we collected dietary records not just once, but three times during the intervention. We did not give specific dietary plans to avoid causing unnatural changes in the participants’ dietary habits. In fact, we were surprised to find such high habitual protein intakes, considering that the well-known recommended dietary allowance of protein for adults is 0.8 g/kg body weight/day. Another possible limitation was that the 10 weeks of the intervention might not have been sufficient for protein supplementation to make a difference.

In conclusion, 10 weeks of Pilates training improved core muscle endurance, joint flexibility, and aspects of body composition in healthy trained women, but these adaptations were not augmented by protein supplementation. Future studies could investigate the effects of longer interventions and also examine different populations, such as untrained individuals of both sexes and individuals with lower habitual protein intake.
